# Supplementary material for: Development and testing of a deep learning algorithm to detect lung consolidation among children with pneumonia using hand-held ultrasound
Source: PLoS One. 2024 Aug 27;19(8):e0309109. doi: 10.1371/journal.pone.0309109 (PMC11349203; doi:10.1371/journal.pone.0309109)
Supplement: S1 Fig — Lung image acquisition areas. Anterior scan areas: right hemithorax–areas 1, 2, 3, and 4; left hemithorax–areas 5, 6, 7, and 8. Posterior scan areas: right hemithorax–areas 9 and 10; left hemithorax–areas 11 and 12. (Created with BioRender.com). (PDF) [file pone.0309109.s001.pdf]

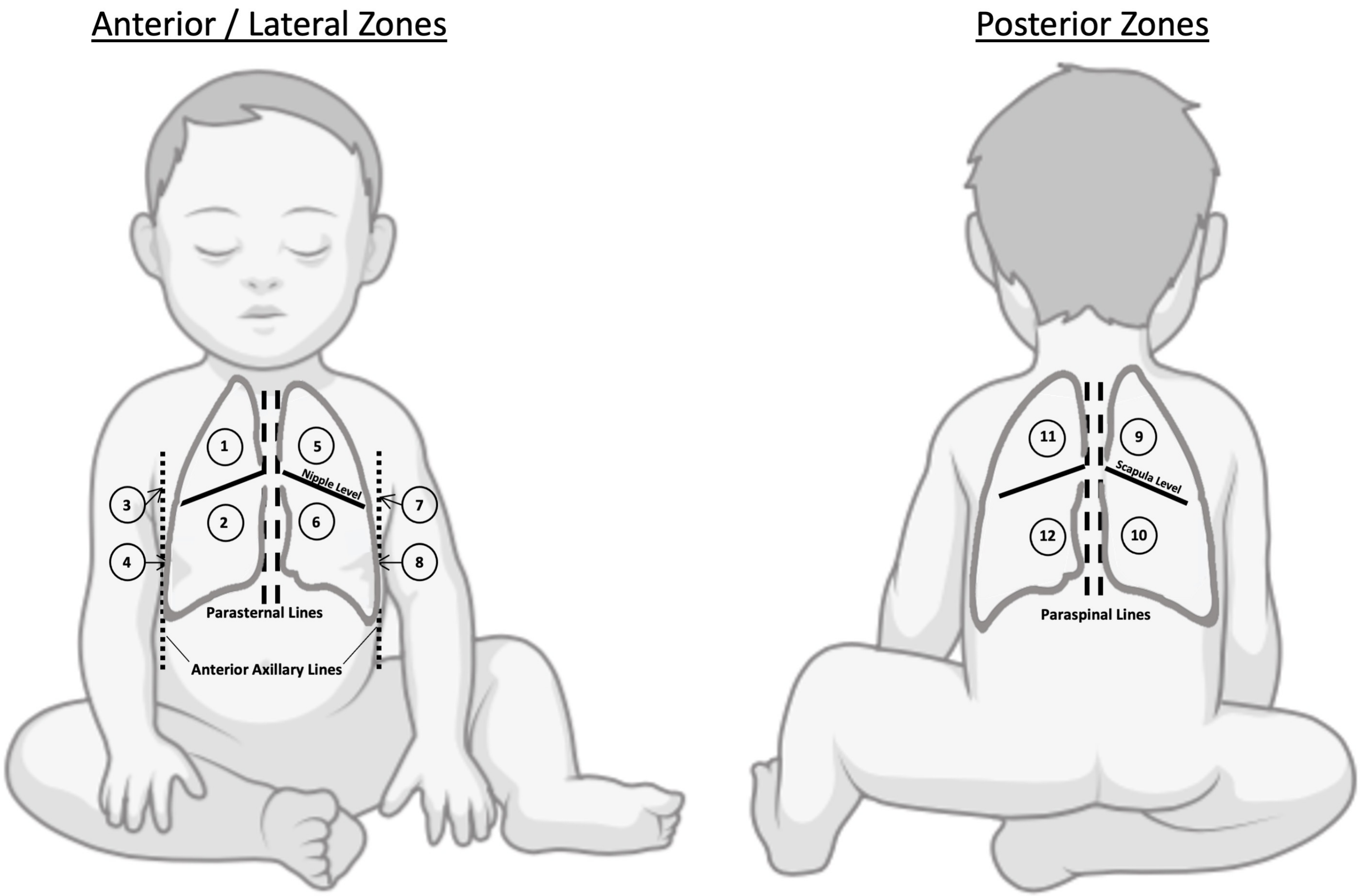

S1 Fig. Scanning zones for Pediatric Lung Ultrasound

Lung image acquisition areas. Anterior scan areas: right hemithorax – areas 1, 2, 3, and 4; left hemithorax – areas 5, 6, 7, and 8. Posterior scan areas: right hemithorax – areas 9 and 10; left hemithorax – areas 11 and 12. (Created with BioRender.com)
