# Supplementary material for: Development and testing of a deep learning algorithm to detect lung consolidation among children with pneumonia using hand-held ultrasound
Source: PLoS One. 2024 Aug 27;19(8):e0309109. doi: 10.1371/journal.pone.0309109 (PMC11349203; doi:10.1371/journal.pone.0309109)
Supplement: Attachment — Submitted filename: LUS_PONE_Response to Reviewers_Apr_29_24.docx [file pone.0309109.s002.docx]

Response to Reviewers:

Re: PONE-D-23-38634, “Development and testing of a deep learning algorithm to detect pediatric pneumonia on a portable ultrasound device”

Reviewer #1:

*1) I agree CXR is the current standard of care, but in meta-analysis lung ultrasound seems to perform better. Perhaps a distinction could be made in this regard:*

We agree that recent meta-analysis comparing lung ultrasound (LUS) to CXR have demonstrated robust diagnostic accuracy in both pediatric and adult populations and have added additional comment and references in the revised Introduction.

*2) The authors mention the use of adult imaging. I understand the focus of your manuscript is on pediatric patients. If possible, I still think the adults you scanned should ideally be included in the description of the study population section. Were adults enrolled with the same criteria?*

Adults 18-80 years old with a clinical diagnosis of pneumonia and CXR and/or chest CT evidence of consolidation were enrolled separately, following provision of written informed consent. Adult patients were excluded if they were intubated, had active sepsis, cardiogenic pulmonary edema, known lung cancer, pulmonary embolus, chronic bronchiectasis or cystic fibrosis, or could not tolerate the LUS procedure. These details have been added to the Methods section of the revised manuscript.

*3) Please consider explaining the rationale for adding adult images in a little more detail. You state it is to “enrich” the data but I think it may be beneficial to explain what about the adult data enriches the AI – for example more studies to train the AI, more generalizability etc.? …I think it could also be an interesting area of discussion in any differences between the adult and pediatric populations as a future consideration but I wouldn’t want this to distract from the focus of your manuscript which I feel is already well written as it is. Perhaps a few sentences could be included commenting on this in the discussion if you feel it could be beneficial. Otherwise, I also understand if you want to keep the focus of the manuscript on children and not comment further.*

We added to the Results section of the revised manuscript additional detail regarding the rational for including in the training data set LUS data from 44 adults hospitalized with pneumonia as follows: “The addition of adult data served the dual purposes of increasing the amount of data (as deep learning networks require large amounts of training data) and adding more diversity. Specifically, the addition of adult data was felt to enhance representation of data from pediatric patients between 14-18 years old in the training data set.”

In our analysis, we did not specifically compare LUS imaging findings between pediatric and adult participants with consolidations. Thus, we feel that adding an additional discussion on age-based differences in identification of consolidations by LUS would not be appropriate for this manuscript.

*4) If you had 117 unique exams, multiplied by 12 that would be 1,404 video clips. You report using 1,193 video clips. I assume the 211 difference related to clips that were not of adequate quality? What were some of the quality issues that made a clip not usable? For example, were their problems with motion artifact or not enough gel? If you could provide exact data regarding clips were rejected that would be interesting if you happened to record it, but I think even just some generalized qualitative description of things that affected quality would be helpful.*

There were 117 unique exams, but the number of videos per exam was quite variable, ranging from 1 to 38. The average number of videos per exam was 10.2. Low quality videos were excluded, with the main issues compromising quality related to poor transducer contact, bad transducer angle, transducer motion, or other organs obstructing visualization of the lung.  An explanation for exclusion of low quality videos is provided in the Results section of the revised manuscript.

*5) Were the images acquired point-of-care at the bedside or in an ultrasound suite or a combination? Were the exams performed by physicians or by sonographers or a combination? Readers may also benefit from a little more information about how the people acquiring the images were trained and what their background was prior to the study. In the discussion you describe “minimally trained research personnel” line 306. I assume the multi-center nature of your study may have individuals with a variety of backgrounds acquiring the imaging? More information about those scanning and how many people scanned would be helpful/interesting if possible.*

All images were acquired point-of-care at the bedside by clinicians or research staff members following training using a study-specific standardized operating procedure; images were not acquired by radiologists or professional sonographers. Prior experience with performance of lung ultrasonography ranged from none to moderate-level of experience. At one pediatric recruitment site, all images were acquired by a single clinician-researcher; at our second pediatric recruitment site, images were acquired by multiple clinician-researchers. Additional details regarding performance of the LUS procedure are included in the Methods section of the revised manuscript.

*6) How many frames per second does Lumify record? Was every single frame labeled?*

The Lumify was operated at 20 frames/second. Every frame of every video containing relevant lung features was annotated. These details are included in the Methods section of the revised manuscript.

*7) My copy of Figure 1 and Figure 2 are grainy.*

We apologize for the poor quality of the uploaded images. We have improved the quality of our figures for the revised manuscript.

Reviewer #2:

1) *Please insert data to clarify the pneumonia diagnosis criteria used for this study*

Following our approved IRB protocols and after receiving written informed consent and assent (if applicable), the medical records from each participant’s relevant hospitalization were reviewed to identify individuals diagnosed with pneumonia. Specifically, the following details have been added to the Methods section of the revised manuscript. “The medical records from each participant’s relevant hospitalization were reviewed to obtain demographic and clinical data and to identify individuals diagnosed with pneumonia. Specifically, data detailing admission and discharge diagnoses, results of any microbiologic testing (e.g., respiratory viral panel results were available for 77% of subjects), performance of pulmonary procedures (eg, chest tube placement; bronchoscopy), and images and clinical radiologists’ interpretation of chest imaging (available for 89% of subjects), were collected.”

*2) Consolidations might be present even in viral pneumonia, please state the relation with etiology*

We agree with the Reviewer that viral infections can be associated with focal pulmonary consolidations. While 77% of the subjects were tested for viral pathogens, our study was not designed or powered to examine correlations between identification of viral pathogens and focal lung consolidations, and we were unfortunately not able to probe this relationship in our data set. We have included this as a limitation in our revised manuscript.

3) *No clear data on relation between dimensions and etiology, even if several paper suggests the relation*

We appreciate the Reviewer’s reference to prior studies demonstrating the capacity of specific LUS findings to delineate different microbiologic (eg viral vs bacterial) causes of lower respiratory tract infection in children. As explained in our response above (Reviewer 2, #2), our study did not include comprehensive testing for causative pathogens and we are unfortunately unable to perform such an analysis with our current data set.

*4) Please provide the explanation for the comparison method, as mentions either CXR or CT scan; there is mentioned that :"Reference standard diagnosis of positive or negative pneumonia was derived from chart data confirming a clinical diagnosis of pneumonia along with consistent findings on radiologic imaging"*

The majority (i.e., 89%) of pediatric participants had CXRs/chest CTs obtained as part of their clinical care. We reviewed their interpretation to ensure that children with a clinical diagnosis of pneumonia had evidence of consolidation on routine clinical imaging.

*5) As pneumonia is not just an imaging diagnosis, maybe the change title should be considered because the study seemed to evaluated just the presence of consolidations*

We agree with the Reviewer that pneumonia is a clinical diagnosis that can be supported by findings of consolidation on lung imaging, and appreciate the suggestion to refine the title of this manuscript. We have retitle the revised manuscript “Development and testing of a deep learning algorithm to detect lung consolidation among children with pneumonia using hand-held ultrasound.”

*6) The study declares that movies with consolidations were evaluated, no data on dimensions, and would be a significant bias, as they suggest etiology*

As detailed above (Reviewer 2, #2 and #3 above), our study was not designed to identify or distinguish the etiology of pneumonia among participating children and this is now included as a study limitation in the revised manuscript.

*7) Small consolidations < 1-1.5 cm are not visible on CXR (considered as gold standard) Berce V, Tomazin M, Gorenjak M, Berce T, Lovrenčič B. The usefulness of lung ultrasound for the aetiological diagnosis of community-acquired pneumonia in children. Sci Rep. 2019. Therefore, the US could be more sensitive in detecting small lesions, please comment on that, how was this considered in your study*

We agree that US may be more sensitive than CXR for detection of small consolidations, and this was a consideration in regard to the algorithm’s false negative and false positive classifications. As discussed in the results section, a quarter of the consolidations missed by the algorithm were smaller in size, while over half of the false positives were triggered by abnormal pleural lines. Our study design, built for initial algorithm development and validation, did not allow us to examine the sensitivity of LUS for small consolidations less than 5 mm. We look forward to performing a study that examines the test characteristics of our LUS protocol and algorithm to address this important consideration. We have, however, added into the Discussion of the revised manuscript the potential for LUS (including the reference from Berce et al) to identify small consolidations.

*8) No data on pleural effusion and no data on empyema or B lines, nor on atelectasis*

The primary goal of this project was to develop an AI algorithm to detect lung consolidations, specifically, among children with a clinical diagnosis of pneumonia using a hand-held ultrasound devise. The dominant lung ultrasound features among this pediatric population were pleural lines, A-lines, B-lines, sub-pleural consolidations and consolidations of different sizes. In this cohort, pleural effusions were present in around 20% of the annotated videos; however, there were insufficient effusion data to allow development of the algorithm for detection of pleural effusions in children.  The presence of empyema, B lines, and suspected atelectasis were not formally analyzed.
