# Supplementary material for: Development and testing of a deep learning algorithm to detect lung consolidation among children with pneumonia using hand-held ultrasound
Source: PLoS One. 2024 Aug 27;19(8):e0309109. doi: 10.1371/journal.pone.0309109 (PMC11349203; doi:10.1371/journal.pone.0309109)
Supplement: Attachment — Submitted filename: PONED2338634 Response to Reviewers.docx [file pone.0309109.s003.docx]

**Response to Reviewers**

PONE-D-23-38634R1

Development and testing of a deep learning algorithm to detect lung consolidation among children with pneumonia using hand-held ultrasound

Review Comments to the Author

Reviewer #1:

1. *Thank you for your revisions and work on this manuscript. The original comments from the review have been addressed.*

Thank you for the valuable feedback and helping to improve our manuscript.

Reviewer #2:

2. *This is an interesting study on AI effect regarding lung consolidation detection in children pneumonia. Please provide data on pneumonia consolidation differentiation from atelectasis , as bronchogram exists in pneumonia and no in atelectasis. This issue must be addressed, in order not to create confusion.*

We thank the reviewer for highlighting that consolidation due to pneumonia must be distinguished from atelectasis, and that the presence of dynamic air bronchograms supports a finding of consolidation resulting from pneumonia.  For this study, all ultrasound images were reviewed and independently labelled by two physician experts in lung ultrasound.  Reviewers separately annotated features of consolidation and atelectasis. The presence of air bronchograms was systematically used to distinguish consolidation resulting from suspected infection (pneumonia), from suspected atelectasis, and images annotated accordingly. For this study only videos that contained consolidation confirmed by two video annotators were included in the positive consolidation set. Suspected atelectasis was not included as a feature of consolidation reflecting pneumonia in training or testing of the model.

We have discussed this in the following sections of the manuscript:

Methods: (line 172) LUS images were annotated for atelectasis

“Labels included…atelectasis…”

Methods: (line 178):

“For this study, only videos that contained consolidation confirmed by two video annotators were included in the positive consolidation set. The presence of air bronchograms was routinely used to distinguish consolidation resulting from suspected infection (pneumonia) from suspected atelectasis with the latter feature deliberately excluded from model training.”

Results: (line 294) Some false positives were attributed to atelectasis

“…the remaining false positives were triggered by atelectasis…”

Limitations (line 362): “…it is not clear how well this algorithm will be able to distinguish pneumonia from other causes of consolidation artifacts on LUS (e.g. atelectasis)

3. *Please provide diagnosis criteria for pneumonia used for the study; there is an addendum: "“The medical records from each participant’s relevant
   hospitalization were reviewed to obtain demographic and clinical data and to identify
   individuals diagnosed with pneumonia. Specifically, data detailing admission and
   discharge diagnoses, results of any microbiologic testing.." but is not clear how did the patients were selected by protocol and criteria used. It looks like diagnosis was made randomly by other physicians, but what were the criteria used; were not established in the study design*

To identify potentially eligible patients for study inclusion, emergency room and hospital ward censuses were reviewed by the study team to identify individuals being evaluated or admitted with suspected pneumonia who met study eligibility requirements, per approved IRB protocols. Following receipt of informed consent (and assent, if appropriate) from eligible participants, LUS was performed. Subsequently, data from the medical records from each participant’s relevant hospitalization were reviewed to obtain demographic and clinical data and to identify individuals diagnosed and treated for pneumonia.  Specifically, data detailing admission and discharge diagnoses, results of any microbiologic testing (respiratory viral panel results were available for 77% of subjects), treatment course, performance of pulmonary procedures (eg, chest tube placement; bronchoscopy), and clinical radiologists’ interpretation of chest imaging (available for 89% of subjects), were collected.

Using this approach, participants with diagnostic studies, clinical course, and documentation supporting a diagnosis of pneumonia were systematically delineated from participants with other disease processes who were not diagnosed with pneumonia (see “Scanning protocol and imaging data” for further details).

As the reviewer notes, the diagnosis of pneumonia is a clinical diagnosis that can include signs such as fever, cough, pleuritic chest pain, and exam findings may include crackles on lung exam with varying degrees of respiratory distress (e.g. tachypnea and/or retractions).

Therefore, our primary definition for pneumonia relied upon our extensive chart review to confirm that a clinical diagnosis of pneumonia was made by treating physicians as evidenced by ICD diagnostic codes for admission and/or discharge diagnosis of pneumonia. We have included further details of this process outline above with edits to the methods in this revision of this manuscript.
